# Infections Caused by* Actinomyces neuii*: A Case Series and Review of an Unusual Bacterium

**DOI:** 10.1155/2016/6017605

**Published:** 2016-02-29

**Authors:** Nathan Zelyas, Susan Gee, Barb Nilsson, Tracy Bennett, Robert Rennie

**Affiliations:** ^1^Provincial Laboratory for Public Health, Walter Mackenzie Health Sciences Centre, University of Alberta Hospital, 8440-112 Street, Edmonton, AB, Canada T6G 2J2; ^2^Queen Elizabeth II Hospital, 10409-98 Street, Grande Prairie, AB, Canada T8V 2E8; ^3^Red Deer Regional Hospital, 3942-50a Avenue, Red Deer, AB, Canada T4N 4E7

## Abstract

*Background. Actinomyces neuii* is a Gram-positive bacillus rarely implicated in human infections. However, its occurrence is being increasingly recognized with the use of improved identification systems.* Objective*. To analyse* A. neuii* infections in Alberta, Canada, and review the literature regarding this unusual pathogen.* Methods*. Cases of* A. neuii* were identified in 2013-2014 in Alberta. Samples were cultured aerobically and anaerobically. A predominant catalase positive Gram-positive coryneform bacillus with no branching was isolated in each case. Testing was initially done with API-CORYNE® (bioMérieux) and isolates were sent to the Provincial Laboratory for Public Health for further testing. Isolates' identities were confirmed by matrix-assisted laser desorption ionization time-of-flight mass spectrometry microbial identification system (MALDI-TOF MS MIS; bioMérieux) and/or DNA sequencing.* Results*. Six cases of* A. neuii* infection were identified. All patients had soft tissue infections; typically, incision and drainage were done followed by a course of antibiotics. Agents used included cephalexin, ertapenem, ciprofloxacin, and clindamycin. All had favourable outcomes.* Conclusions*. While* A. neuii* is infrequently recognized, it can cause a diverse array of infections. Increased use of MALDI-TOF MS MIS is leading to increased detection; thus, understanding the pathogenicity of this bacterium and its typical susceptibility profile will aid clinical decision-making.

## 1. Introduction

Members of the genus* Actinomyces* are the commonest cause of the oft-described disease, actinomycosis. This clinical syndrome is characterized by its chronic course, the invasion of multiple tissue planes, the formation of sinus tracts and masses resembling malignancy, the production of “sulfur granules,” and its relapsing nature when short courses of antimicrobials are used [1–3]. Although actinomycosis typically involves cervicofacial, thoracic, and abdominal regions,* Actinomyces* species can cause a variety of infections, including abscesses and skin infections in various locations [4–6], ocular infections [5], urinary tract infections [4, 6], genital infections [4, 6], intrauterine contraceptive device infections [5, 7], appendicitis [4], cholecystitis [4], osteomyelitis [5, 6], bacteremia [4–6], endocarditis [8, 9], CNS infections [10–13], and many others [14].

Organisms belonging to the* Actinomyces* genus are typically aerotolerant Gram-positive rods that grow in branching filaments. Isolates are usually catalase and urease negative, CAMP negative, and nonpigmented and produce succinic and lactic acid as major end products of metabolism [15]. However, not all* Actinomyces* species share all of these characteristics, and one of these is the clinically relevant* Actinomyces neuii*.

Prior to 1994,* A. neuii* isolates were classified as members of CDC (Centers for Disease Control) fermentative coryneform group 1 or group 1-like [16, 17]. These isolates were common in that they were Gram-positive coryneform rods, nonmotile, catalase positive, urease negative, esculin hydrolysis negative, CAMP positive, and fermented glucose, maltose, sucrose, mannose, and xylose [16, 17]. Sequencing of the 16S rRNA genes of CDC coryneform group 1 and group 1-like isolates revealed that they are closely related subspecies belonging to the* Actinomyces* genus:* A. neuii* subsp.* neuii* and* A. neuii* subsp.* anitratus* [18]. The term “neuii” was chosen to honour Dr. Harold Neu, a well-known physician of infectious diseases; “anitratus” was chosen to reflect the lack of nitrate reduction by* A. neuii* susp.* anitratus* [18].

Since its classification,* A. neuii* has been implicated in a number of human infections [19]. However, due to its unusual laboratory characteristics, isolates have likely been dismissed as members of the* Corynebacterium* genus and its occurrence in clinical specimens is probably underreported. Indeed, the use of advanced bacterial identification systems, such as matrix-assisted laser desorption ionization time-of-flight mass spectrometry microbial identification systems (MALDI-TOF MS MIS), in clinical laboratories will likely result in an increased frequency of correctly identifying bacteria that were previously difficult to identify using traditional phenotypic methods [20, 21]. Because of the increasingly widespread use of MALDI-TOF MS MIS, understanding the significance of isolating previously underrecognized pathogens is important in guiding laboratory reporting and clinical practices.

The present study examines cases of* A. neuii* infections detected in several community and tertiary care laboratories and confirmed by a reference microbiology laboratory. The literature regarding* A. neuii* infections is reviewed and compared to the findings in this series of cases.

## 2. Methods

Clinical specimens were submitted to local microbiology laboratories during 2013-2014 in Alberta, Canada. Samples were initially examined microscopically after Gram staining and cultured aerobically and, if deemed appropriate, anaerobically on typical culture media including sheep blood agar, brain heart infusion agar, and phenylethyl alcohol agar (Dalynn Biologicals Inc., Calgary, Alberta). Isolates were initially identified by producing characteristic convex, circular, smooth, and white colonies with entire edges with consistent Gram stain appearance, catalase reaction, and major acid end-product profiles as determined by gas-liquid chromatography. If the specimen was submitted and initially worked up by a local microbiology laboratory, isolates suspected of being* A. neuii* were further identified using the API-CORYNE (bioMérieux, France) test strip. Further confirmation of isolates' identities was performed at the Alberta Provincial Laboratory for Public Health, Edmonton, Alberta, using the VITEK® MS MALDI-TOF MS MIS (bioMérieux, France) and/or 16S rRNA gene sequencing. The VITEK MS MALDI-TOF MS MIS assay was carried out according to the manufacturer. The* in vitro* diagnostic database used for comparing spectra was VITEK MS v2.0 Knowledge Base (bioMérieux, France); matches with a ≥99.9% confidence were accepted as* A. neuii*.

In order to carry out the sequencing of a portion of the 16S rRNA gene, genomic DNA was extracted using a pure culture of organism growing on sheep blood agar incubated anaerobically at 35°C. A sweep of organism was suspended in 12 mM Tris buffer, pH 7.4, and centrifuged at 13,000 ×g for 3 minutes. The pellet was resuspended in rapid lysis buffer (100 mM NaCl; 10 mM Tris-HCl, pH 8.3; 1 mM EDTA, pH 9.0; 1% Triton X-100) and subjected to three freeze/thaw cycles of −80°C for 15 minutes followed by complete thawing. This preparation was then boiled for 15 minutes, cooled to room temperature, and centrifuged at 13,000 ×g for 15 minutes. Nucleic acid in the supernatant was used for conventional polymerase chain reaction (PCR) with the following previously described 16S rRNA gene primers: 16S-F 5′AGA GTT TGA TCA TGG CTC AG 3′ and 16S-R 5′GGA CTA CCA GGG TAT CTA AT 3′ [46]. Amplification reactions were carried out using Platinum®* Pfx* DNA Polymerase (Thermo Fisher Scientific, Waltham, MA, USA) according to the manufacturer with the following cycling conditions: 94°C for 2 minutes, then 25 cycles of 94°C for 15 seconds, 50°C for 30 seconds, and 68°C for 20 seconds. After product cleanup and quantification with the ChargeSwitch® Kit and the NanoDrop*™* 2000 (Thermo Fisher Scientific), respectively, DNA sequencing was performed using the BigDye® Cycle Sequencing and BigDye XTerminator*™* Purification Kits (Thermo Fisher Scientific) on the Applied Biosystems*™* 3500xL Genetic Analyzer (Thermo Fisher Scientific). Sequence data was viewed and edited using the BioEdit program and uploaded to the Ribosome Database Project to obtain sequence matches [47]. All but one of the sequences obtained were over 350 nucleotides; despite multiple attempts, one amplicon yielded only 257 nucleotides of high quality sequence (see Supplementary Data in the Supplementary Material available online at http://dx.doi.org/10.1155/2016/6017605). In each case, a seqmatch (S_ab) score of 1.000 (signifying 100% identity) was found with at least one other submitted* A. neuii* rRNA gene nucleotide sequence. Identification to the subspecies level was not performed.

Susceptibility testing was carried out using gradient diffusion Etest® strips (bioMérieux, France) under the conditions described for anaerobic organisms by the Clinical and Laboratory Standards Institute (CLSI; [48]). Briefly, a 0.5 McFarland suspension of organism was inoculated onto Anaerobic Laked Blood Agar (Dalynn Biologicals Inc.) and incubated at 36°C anaerobically for 48 hours. Minimal inhibitory concentrations (MICs) were determined as per the manufacturer's instructions. Breakpoints were interpreted as per CLSI guidelines for anaerobic bacteria [48]. Patient information was obtained from specimen requisitions and the treating physicians. The work described in this paper does not require ethics review as defined in Section 2.4 of the Tri-Council Policy Statement: Ethical Conduct for Research Involving Humans [49].

## 3. Results

*Actinomyces neuii* was isolated from eight patients from February 2013 to July 2014; patient information was available for six of these cases (Table 1). From each patient, a predominant Gram-positive, catalase positive coryneform bacillus was isolated. Gas chromatography revealed a major succinic acid peak for the isolates. In all three cases where the isolate was initially worked up by a local laboratory, the API-CORYNE test strip correctly identified the organism as* A. neuii*. The first five isolates were confirmed as* A. neuii* using 16S rRNA gene sequencing; this allowed the laboratory's VITEK MS MALDI-TOF MS MIS instrument to be validated for identifying* A. neuii* and therefore be used without further extensive confirmatory testing. Patient ages ranged from 30 to 85 years; all but one of the patients were male. There was no obvious trend in patient comorbidities. While one of the specimens was collected from a lower extremity skin ulcer, the remaining five were collected from abscesses in different locations. Gram smears usually revealed the presence of multiple types of bacteria in the clinical specimens and the resulting cultures grew at least two different bacterial species as coisolates in all but one case (Table 1). The most common coisolates were anaerobic bacteria, but the more common skin and soft tissue infectious agent,* Staphylococcus lugdunensis*, was isolated in two cases. Susceptibility testing showed that all of the* A. neuii* isolates had low minimal inhibitory concentrations to penicillin, broader spectrum *β*-lactams, and vancomycin; one isolate had an MIC ≥ 256 mg/L to clindamycin while the others had MICs of ≤0.032 mg/L. Treatment of the abscesses usually involved incision and drainage followed by seven- to 18-day courses of a *β*-lactam, though one of the abscesses was treated with a four-week course of ciprofloxacin without drainage. None of the patients were noted to have relapses following therapy.

## 4. Discussion

*Actinomyces neuii* has been isolated in a wide range of clinical scenarios but has rarely been reported in the literature. There are less than 100 cases reported in the literature of* A. neuii* infections; we and others have hypothesized that dismissal as a commensal coryneform bacillus may be the reason. While there have only been scattered reports of* A. neuii* causing infection since interest in the organism was originally raised in the mid-1990s, the frequency of reports recognizing it as a pathogen has recently increased likely due to the use of advanced identification systems. Abscesses are the most common manifestation of* A. neuii* infection with more than half of the reported cases describing abscesses or infected atheromas (Table 2) [6, 17, 19, 22–45]. A number of other infectious entities implicating* A. neuii* have been described, including ulcer infections, cellulitis, urinary tract infections, and prostatitis (Table 2).

There have been two reported cases of* A. neuii* chorioamnionitis, one of which resulted in neonatal sepsis after premature delivery [19, 27]. In the latter case, the organism was isolated from multiple sites from the neonate, including the external ear canal, a gastric aspirate, and blood [27].

As demonstrated by the above case,* A. neuii* can also cause more invasive infections. There are multiple other cases of* A. neuii* bacteremia, various prostheses infections, two cases of chronic osteomyelitis, two cases of peritoneal dialysis-associated peritonitis, and single cases of native aortic valve endocarditis, chronic pericarditis, and lymphadenitis; interestingly, there have been multiple reports of* A. neuii* causing endophthalmitis, the majority of which occurred postoperatively (Table 2).

Intriguingly, there has been only one case reported of* A. neuii* causing a classical clinical picture of actinomycosis with recurrent breast abscesses, fistula formation, filamentous growth, and sulfur granules [23]. This is in agreement with the findings of our study, as none of the patients exhibited such findings nor did they require extended durations of antibiotic therapy.

The infections caused by* A. neuii* described in the present study were all associated with abscess formation in soft tissue except for one, which was isolated from an ankle ulcer. The latter case may not actually represent an infection, as there were no white cells in the clinical specimen. The age range of patients infected with* A. neuii* in the literature is 0–94 years with a mean of 50 years; this is comparable with the ages of patients in this study. While reports in the literature show an almost even number of males and females acquiring* A. neuii* infections, the majority of the patients in our study were male; this is likely due to the small number of cases examined.

Previous studies found that abscesses typically included mixed anaerobic organisms and skin flora as coisolates (Table 2). This is similar to the present study in which four of six specimens grew anaerobic bacteria and one grew coagulase-negative staphylococci; as well, the patient from whom* A. neuii* was isolated in pure culture had Gram-positive cocci seen in the direct smear, making it possible that this was also a mixed anaerobic infection from which at least one anaerobe failed to grow in culture. However, this study also had a larger variety of aerobic coisolates in specimens than was reported in previous studies. It is difficult to assess the specific role of* A. neuii* in these mixed infections, but its presence in polymicrobial abscesses makes up the vast majority of cases. Improved identification of* A. neuii* in the laboratory will undoubtedly help to clarify its role in disease.

There are few studies describing the use of MALDI-TOF MS MIS in identifying* A. neuii*. One other study showed that the VITEK MS system was able to correctly identify* A. neuii* to the species level in eight of 12 instances [21]. Three groups used Bruker MALDI-TOF MS MIS (Bruker, Billerica, MA, USA); two isolates were designated as* A. neuii* with a “secure genus, probable species identification” level of confidence [20, 30], while the degree of certainty in identification of the other isolate was not described [45]. Due to the limited amount of experience with either type of MALDI-TOF MS MIS, there is no current evidence that one system is superior to the other in identifying* A. neuii*.

The majority of tested isolates show susceptibility to *β*-lactams including penicillin G, ampicillin, cefazolin, cefuroxime, ceftriaxone, and imipenem [17, 19, 27, 28, 34–36, 40, 42, 43]. Other agents with* in vitro* activity against* A. neuii* include clindamycin, erythromycin, tetracycline, and vancomycin [17, 19, 27, 28, 35, 36, 40, 42]. Fluoroquinolones (including ciprofloxacin and levofloxacin), aminoglycosides (including gentamicin and amikacin), and trimethoprim-sulfamethoxazole frequently demonstrate less* in vitro* activity against isolates [19, 35, 36, 40].

Similar to previous studies, the isolates in the current series were generally quite susceptible to the tested antibiotics. All isolates had low MICs to penicillin, amoxicillin-clavulanate, and vancomycin; only one isolate showed resistance to clindamycin.

As shown in Table 2, treatment regimens in the literature are varied and have been dictated by the type and severity of infection. In general, successful treatment regimens often involved the use of *β*-lactam antibiotics as well as appropriate surgical interventions. This was also the case in the present study, as almost all of the treatments involved incision and drainage of abscesses with subsequent *β*-lactam courses.

While* A. neuii* is not a commonly identified organism, there are numerous reports of it causing both invasive and noninvasive disease. Fortunately, it is a very susceptible organism that can often be treated with *β*-lactams following drainage of abscesses or other surgical management. Understanding its clinical significance and typical susceptibility patterns will ease decision-making when the organism is encountered, as it surely will be with the current widespread use of MALDI-TOF MS MIS.

## Supplementary Material

Supplementary data: 16S rRNA gene segment sequences used for the confirmation of *Actinomyces neuii* identification.

## Figures and Tables

**Table 1:** Characteristics of patients from which *A. neuii* was detected and susceptibility profiles of isolates.

Age, sex	Comorbidities^a^	Infection	Gram smear^b^	Coisolates	MICs of *A. neuii* isolates (mg/L)^c^	Treatment
30, M	Previous head injury, right ACL repair	Left thigh abscess	4+WBC 4+GPC 4+GPB	*Prevotella bivia* Anaerobic GPC	Penicillin G ≤0.016 Amox/clav 0.016 Imipenem 0.023 Vancomycin 0.5 Clindamycin ≥256	Incision and drainage Cephalexin 7 days

45, M	TIIDM, dyslipidemia	Left inguinal abscess	3+WBC 2+GPC 2+GNB 1+GPB	*Staphylococcus lugdunensis* Anaerobic GPC	Penicillin G ≤0.016 Amox/clav 0.016 Imipenem 0.023 Vancomycin 0.5 Clindamycin ≤0.016	Incision and drainage Cephalexin 7 days

46, M	Paraplegia, renal calculi, atrial fibrillation, previous endocarditis, sacral ulcer	Right axillary abscess	3+WBC 3+GPB 2+GPC	None	Penicillin G ≤0.03 Amox/clav ≤0.016 Imipenem 0.023 Vancomycin ≤0.5 Clindamycin ≤0.016	Incision and drainage Cephalexin (unknown duration)

48, M	Hypertension, TIIDM, obesity, previous Fournier's gangrene, diabetic foot infections	Right groin abscess	3+WBC 3+GPB 3+GNB 2+GPC	*Proteus mirabilis* *Staphylococcus lugdunensis* *Actinomyces* sp. Mixed anaerobes	Penicillin G 0.006 Amox/clav ≤0.016 Imipenem 0.023 Vancomycin 0.5 Clindamycin 0.023	Incision and drainage Ceftriaxone 3 days Ertapenem 5 days Amox/clav 10 days

68, M	Bilateral spermatoceles with spermatocelectomies	Postoperative right scrotum abscess	2+WBC 3+GPC 2+GPB 2+GNB	Coagulase-negative *Staphylococcus* Coryneform bacillus *Propionibacterium* sp.	Penicillin G ≤0.016 Amox/clav ≤0.016 Imipenem 0.032 Vancomycin 0.5 Clindamycin 0.032	Ciprofloxacin 28 days

85, F	Hypertension, hypothyroidism, celiac disease	Postbiopsy left ankle ulcer	3+GNB 2+GPC	*Acinetobacter baumannii*/*calcoaceticus* complex *Streptococcus agalactiae*	Penicillin G ≤0.06 Amox/clav ≤0.016 Imipenem 0.023 Vancomycin 0.38 Clindamycin 0.023	Wound care Clindamycin 10 days Cephalexin 7 days

^a^TIIDM, type II diabetes mellitus; ACL, anterior cruciate ligament.

^b^WBC, white blood cells; GPC, Gram-positive cocci; GPB, Gram-positive bacilli; GNB, Gram-negative bacilli; for WBC, 1+ represents <1 cell per high power field (×100 oil immersion lens), 2+ represents 1–5 cells, 3+ represents 6–10 cells, and 4+ represents >10 cells; for bacteria, 1+ represents ≤1 cell per high power field, 2+ represents 2–10 cells, 3+ represents 11–50 cells, and 4+ represents >50 cells.

^c^MIC, minimal inhibitory concentration; amox/clav, amoxicillin-clavulanate.

**Table 2:** Characteristics of previously reported cases of *A. neuii* infections in the literature.

Infection	Number of cases	Coisolates	Treatment	Outcomes	References
Abscess/infected atheroma^a^	56	Coagulase-negative staphylococci *Enterococcus* spp. *Corynebacterium* spp. Anaerobes	Incision and drainage, with or without antibiotic therapy (usually *β*-lactams, occasionally tetracycline, ciprofloxacin)	Generally favourable if source control is achieved	[6, 17, 19, 22–26]

Cutaneous infection^b^	9	Coagulase-negative staphylococci (eight cases)	Not reported	Favourable	[17, 19]

Genitourinary infection^c^	6	None	Antibiotic therapy with *β*-lactams	Favourable	[19, 27]

Bacteremia^d^	8	None	Two cases reported therapy: Ampicillin with gentamicin (two weeks), followed by PO penicillin (four weeks) Initial ciprofloxacin followed by imipenem^e^	Generally favourable (one mortality)	[19, 27]

Endocarditis	1	None	Aortic valve excision with homograft implantation followed by ampicillin (three weeks), ceftriaxone (nine weeks), and then PO doxycycline (nine months)	Favourable	[28]

Chronic pericarditis	1	None	Pericardial fluid drainage and antibiotic therapy (specific antibiotics not reported)	Not reported	[29]

Lymphadenitis	1	Viridans group streptococci *Prevotella timonensis* Anaerobic Gram-positive cocci	Lymph node and fistula excision followed by IV and then PO amoxicillin-clavulanate (six months)	Favourable	[30]

Osteomyelitis	2	*Dermabacter hominis* (one case)	Surgical curettage followed by *β*-lactam therapy for multiple months	Favourable	[25, 31]

Peritonitis (secondary to peritoneal dialysis)	2	None	Catheter retention with either intraperitoneal cefazolin and ceftazidime (two weeks) followed by penicillin G (four weeks), or intraperitoneal ampicillin, teicoplanin, and tobramycin (two weeks)	Favourable	[32, 33]

Endophthalmitis	6	None	Various systemic (PO or IV) and direct (intravitreal or subconjunctival injections, drops) antibiotics	Favourable	[34–38]

Prosthetic material infection^f^	8	Typically none Coagulase-negative staphylococci and mixed anaerobes (breast implant case)	Removal/replacement of prosthetic material followed by prolonged antibiotic therapy (weeks to one year, depending on the infection)	Favourable	[25, 39–45]

^a^Including breast, axillary, inguinal, iliac crest, ischiorectal, and pilonidal abscesses; one case of hidradenitis suppurativa; most sites were not specified.

^b^Including ulcer infections, diabetic foot ulcer infections, and cellulitis.

^c^Including urinary tract infections, prostatitis, and chorioamnionitis.

^d^Including one case of neonatal sepsis secondary to chorioamnionitis; the remaining cases had unclear or unreported sources.

^e^The patient treated with this regimen is the single mortality reported in the literature associated with* A. neuii *infection.

^f^Including infections of an intravenous catheter, a mechanical heart valve, a hip prosthesis, a penile prosthesis, breast implants, and ventriculoperitoneal shunts.
