# Supplementary material for: Infections Caused by Actinomyces neuii: A Case Series and Review of an Unusual Bacterium
Source: Can J Infect Dis Med Microbiol. 2016 Feb 29;2016:6017605. doi: 10.1155/2016/6017605 (PMC4904567; doi:10.1155/2016/6017605)
Supplement: Supplementary file 1 — Supplementary data: 16S rRNA gene segment sequences used for the confirmation of Actinomyces neuii identification. [file 6017605.f1.pdf]

Infections caused by *Actinomyces neuii*: a case series and review of an unusual bacterium

Supplementary data

16S rRNA gene segment sequences used for the confirmation of *A. neuii* identification

```
>sequence 1
GGGATCCATTGGTGCTTGCACTTTTGGTGAGAGTGGCGAACGGGTGAGTA
ACACGTGAGTAACCTGCCCTTTTCTTTGGGATAAGCCTGGGAAACTGGGT
CTAATACTGGATGTTCCGGCTTCCTCGCATGGGGTTGTTGGGAAAGGTTT
TTTCTGGATTGGGATGGGCTCGCGGCTTATCAGCTTGTTGGTGGGGTGAT
GGCTTACCAAGGCTTTGACGGGTAGCCGGCCTGAGAGGGTGGTCGGTCAC
ACTGGGACTGAGATACGGCCCAGACTCCTACGGGAGGCAGCAGTGGGGGA
TATTGCACAATGGACGCAAGTCTGATGCAGCGACGTCGTGTGGGGGATGA
AGGCCTTCGGGTTGTAAACTCCTTTCGCCTGCGGAAAAGGCAGGGTTTTG
GCCTTGTTGATGGTAGTGGGTAAAGAAGCGCCGGCTAACTACGTGCCAGC
AGCCGCGGTAATACGTAGGGCGCGAGCGTTGTCCGGAATTATTGGGCGTA
AAGGGCTCGTAGGCGGTTTGTCGCGTCTAGCGTTTAAGGCTCGGGCTTAA
CCCGGGTTTGCGTTGGGTACGGGCAGGCTTGAGTGCGGTAGGGGTAACTG
GAATTCCTGGTGTAGCGGTGGAATGCGCAGATATCAGGAGGAACACCGGT
GGCGAAGGCGGGTTACTGGGCCGTTACTGACGCTGAGGAGCGAGAGCGT


>sequence 2
GTGCTTGCACTTTTGGTGAGAGTGGCGAACGGGTGAGTAACACGTGAGTA
ACCTGCCCTTTTCTTTGGGATAAGCCTGGGAAACTGGGTCTAATACTGGA
TGTTCCGGCTTCCTCGCATGGGGTTGTTGGGAAAGGTTTTTTCTGGATTG
GGATGGGCTCGCGGCTTATCAGCTTGTTGGTGGGGTGATGGCTTACCAAG
GCTTTGACGGGTAGCCGGCCTGAGAGGGTGGTCGGTCACACTGGGACTGA
GATACGGCCCAGACTCCTACGGGAGGCAGCAGTGGGGGATATTGCACAAT
GGACGCAAGTCTGATGCAGCGACGTCGTGTGGGGGATGAAGGCCTTCGGG
TTGTAAACTCCTTTCGCCTGCGGAAAAGGCAGGGTTTTGGCCTTGTTGAT
GGTAGTGGGTAAAGAAGCGCCGGCTAACTACGTGCCAGCAGCCGCGGTAA
T


>sequence 3
CTAATACTGGATGTTCCGGCTTCCTCGCATGGGGTTGTTGGGAAAGGTTT
TTTCTGGATTGGGATGGGCTCGCGGCTTATCAGCTTGTTGGTGGGGTGAT
GGCTTACCAAGGCTTTGACGGGTAGCCGGCCTGAGAGGGTGGTCGGTCAC
ACTGGGACTGAGATACGGCCCAGACTCCTACGGGAGGCAGCAGTGGGGGA
TATTGCACAATGGACGCAAGTCTGATGCAGCGACGTCGTGTGGGGGATGA
AGGCCTTCGGGTTGTAAAC


>sequence 4
GTGTTTTTGGTGAGAGTGGCGAACGGGTGAGTAACACGTGAGTAACCTGC
CCTTTTCTTTGGGATAAGCCTGGGAAACTGGGTCTAATACTGGATGTTCC
GGCTTCCTCGCATGGGGTTGTTGGGAAAGGTTTTTTCTGGATTGGGATGG
GCTCGCGGCTTATCAGCTTGTTGGTGGGGTGATGGCTTACCAAGGCTTTG
ACGGGTAGCCGGCCTGAGAGGGTGGTCGGTCACACTGGGACTGAGATACG
GCCCAGACTCCTACGGGAGGCAGCAGTGGGGGATATTGCACAATGGACGA
AAGTCTGATGCAGCGACGTCGTGTGGGGGATGAAGGCCTTCGGGTTGTAA
ACTCCTTTCGCCCGCGGAAAAGGCAGGGTTTTGGCCTTGTTGATGGTAG
```

>sequence 5
TCGAACGGGATCCATTGGTGCTTGCACTTTTGGTGAGAGTGGCGAACGGG
TGAGTAACACGTGAGTAACCTGCCCTTTTCTTTGGGATAAGCCTGGGAAA
CTGGGTCTAATACTGGATGTTCCGGCTTCCTCGCATGGGGTTGTTGGGAA
AGGTTTTTTCTGGATTGGGATGGGCTCGCGGCTTATCAGCTTGTTGGTGG
GGTGATGGCTTACCAAGGCTTTGACGGGTAGCCGGCCTGAGAGGGTGGTC
GGTCACACTGGGACTGAGATACGGCCCAGACTCCTACGGGAGGCAGCAGT
GGGGGATATTGCACAATGGACGCAAGTCTGATGCAGCGACGTCGTGTGGG
GGATGAAGGCCTTCGGGTTGTAAACTCCTTTCGCCTGCGGAAAAGGCAGG
GTTTTGGCCTTGTTGATGGTAGTGGGTAAAGAAGCGCCGGCTAACTACGT
GCCAGCAGCCGCGGTAATACGTAGGGCGCGAGCGTTGTCCGGAATTATTG
GGCGTAAAGGGCTCGTAGGCGGTTTGTCGCGTCTAGCGTTTAAGGCTCGG
GCTTAACCCGGGTTTGCGTTGGGTACGGGCAGGCTTGAGTGCGGTAGGGG
TAACTGGAATTCCTGGTGTAGCGGTGGAATGCGCAGATATCAGGAGGAAC
ACCGGTGGCGAAGGCGGGTTACTGGGCCGTTACTGACGCTGAGGAGCGAG
A
